# Stereotactic EEG-guided radiofrequency thermocoagulation versus anterior temporal lobectomy for mesial temporal lobe epilepsy with hippocampal sclerosis: study protocol for a randomised controlled trial

**DOI:** 10.1186/s13063-021-05378-3

**Published:** 2021-06-29

**Authors:** Yi-He Wang, Si-Chang Chen, Peng-Hu Wei, Kun Yang, Xiao-Tong Fan, Fei Meng, Jia-Lin Du, Lian-Kun Ren, Yong-Zhi Shan, Guo-Guang Zhao

**Affiliations:** 1 Department of Neurosurgery, Xuanwu Hospital, Capital Medical University, No. 45, Changchun Street, Xuanwu District, Beijing, 100053 China; 2 Department of Evidence-based Medicine, Xuanwu Hospital, Capital Medical University, Beijing, 100053 China; 3 Department of Neurology, Xuanwu Hospital, Capital Medical University, Beijing, 100053 China; 4 Center of Epilepsy, Beijing Institute for Brain Disorder, Beijing, 100069 China

**Keywords:** Mesial temporal lobe epilepsy, Stereotatic electroencephalograph (SEEG)-guided radiofrequency thermocoagulation (RF-TC), Randomised controlled trial

## Abstract

**Introduction:**

In this report, we aim to describe the design for the randomised controlled trial of Stereotactic electroencephalogram (EEG)-guided Radiofrequency Thermocoagulation versus Anterior Temporal Lobectomy for Mesial Temporal Lobe Epilepsy with Hippocampal Sclerosis (STARTS). Mesial temporal lobe epilepsy (mTLE) is a classical subtype of temporal lobe epilepsy that often requires surgical intervention. Although anterior temporal lobectomy (ATL) remains the most popular treatment for mTLE, accumulating evidence has indicated that ATL can cause tetartanopia and memory impairments. Stereotactic EEG (SEEG)-guided radiofrequency thermocoagulation (RF-TC) is a non-invasive alternative associated with lower seizure freedom but greater preservation of neurological function. In the present study, we aim to compare the safety and efficacy of SEEG-guided RF-TC and classical ATL in the treatment of mTLE.

**Methods and analysis:**

STARTS is a single-centre, two-arm, randomised controlled, parallel-group clinical trial. The study includes patients with typical mTLE over the age of 14 who have drug-resistant seizures for at least 2 years and have been determined via detailed evaluation to be surgical candidates prior to randomisation. The primary outcome measure is the cognitive function at the 1-year follow-up after treatment. Seizure outcomes, visual field abnormalities after surgery, quality of life, ancillary outcomes, and adverse events will also be evaluated at 1-year follow-up as secondary outcomes.

**Discussion:**

SEEG-guided RF-TC for mTLE remains a controversial seizure outcome but has the advantage for cognitive and visual field protection. This is the first RCT studying cognitive outcomes and treatment results between SEEG-guided RF-TC and standard ATL for mTLE with hippocampal sclerosis. This study may provide higher levels of clinical evidence for the treatment of mTLE.

**Trial registration:**

ClinicalTrials.gov NCT03941613. Registered on May 8, 2019. The STARTS protocol has been registered on the US National Institutes of Health. The status of the STARTS was recruiting and the estimated study completion date was December 31, 2021.

## Background

Drug-resistant epilepsy is defined as regular intake of more than one first-line antiepileptic drug (AED) for more than 2 years without adequate seizure control [1]. Frequent seizures severely impact the quality of life and may also lead to cognitive deterioration. As the most common and best-characterised electro-clinical epilepsy syndrome, mesial temporal lobe epilepsy (mTLE) accounts for approximately 40% of epilepsy cases in adults [2]. TLE refers to syndromes in which the temporal lobe is involved in the generation or conduction of the epileptic network [3, 4]. In patients with mTLE, epileptic activity originates from the mesial structures of the unilateral temporal lobe, including the hippocampus, amygdala, and parahippocampal gyrus. First described in the nineteenth century, hippocampal sclerosis (i.e. neuronal loss and gliosis) is the most common pathology in patients with mTLE [5].

In 1950, Penfield and Flanigin described anterior temporal lobectomy (ATL) for the treatment of temporal lobe seizures [6]. After more than half a century of development, ATL has become the standard treatment for mTLE, achieving nearly 80% seizure freedom in the long term (i.e. after 5 years) [4, 7, 8]. However, ATL is associated with complications such as visual field defects, memory loss, and emotional disturbances, which can decrease the quality of life following the procedure [9, 10]. In addition, approximately 20% of patients experience recurrent seizures to varying degrees, which may be attributable to inaccurate localisation of the epileptogenic zone or incomplete surgical resection [8].

Radiofrequency thermocoagulation (RF-TC) was first utilised for patients with mTLE in 1980 [11]. However, given the limitations of imaging technology and stereotactic techniques at that time, it was associated with lower rates of seizure freedom than ATL [12–14]. Recent advancements in multi-modal imaging technology and the development of frameless, robotic stereotactic assistance systems have dramatically improved RF-TC, which can now be guided by stereotactic electroencephalography (SEEG), helping to localise and target the epileptogenic zone [14–16]. Although thousands of patients have undergone RF-TC, the results of this procedure for mTLE remain controversial.

Several recent studies have reported that radiofrequency ablation or RF-TC for mTLE may lead to long-term (at least 1 year) seizure-freedom rates of 25–70% [12, 14, 17–19]. Furthermore, case series from various countries have demonstrated the cognitive benefits of SEEG-guided RF-TC [20]. Optimisation of SEEG strategies can increase the seizure-freedom rate to 76.2% at the 1-year follow-up, with approximately 5% of patients experiencing cognitive impairment at this stage (unpublished data from our series). However, similar to findings reported in previous studies, cognitive function decreased by 25% 1 year after ATL in our group (unpublished data from our series). Thus, given its ability to preserve cognitive function, we believe that SEEG-guided RF-TC can be used as a first-line or complementary treatment for mTLE. However, randomised studies are required to verify this hypothesis.

In our present study (STARTS, *ClinicalTrials.gov: NCT03941613, registered on May 8, 2019*), we aim to investigate the safety and efficacy of SEEG-guided RF-TC for the treatment of mTLE. In this single-centre randomised controlled trial (RCT), we will compare RF-TC as a control treatment with ATL in order to determine the optimal strategy for patients with mTLE.

## Methods/design

### General study design

STARTS is a single-centre, two-arm, randomised controlled, parallel-group clinical trial. The study will enrol patients with refractory mTLE, who will be followed up for 1 year after treatment. Primary outcome measures will include intelligence quotient. Secondary outcome measures will include seizure outcome, visual field, quality of life, average hospitalisation expenses, and adverse events.

### Participant selection

The study aims to enrol male or female participants ranging in age from 14 to 65 years who have had drug-resistant seizures for at least 2 years and who have been determined via detailed evaluation to be candidates for surgery prior to randomisation. Patients will be evaluated based on seizure history and mTLE semiology from outpatient and multidisciplinary team (MDT). Pre-surgical examinations will be carried out before enrolment. The following eligibility criteria have been adopted:

1. Normalised treatment with at least one or more first-line antiepileptic drugs (AEDs) for more than 2 years with still seizure attack. Appropriate doses must have been administered, and treatment must have failed due to inefficacy rather than intolerance
2. Persistence of disabling seizures (at least three times per 3 months or greater) and at least one or more seizures in the preceding month
3. Age ≥ 14 years at enrolment
4. Simple and complex partial seizures, with or without secondary generalised seizures beginning in childhood or later, with or without prior febrile convulsions
5. Auras occurring in isolation that are not primary sensory in nature (other than olfactory or gustatory)
6. Intelligence quotient (I.Q.) greater than 70
7. Hippocampal atrophy on T1-weighted magnetic resonance imaging (MRI) with increased ipsilateral mesial signal on T2-weighted and Flair MRI
8. Interictal EEG showing focal or lateralised spikes over the temporal, frontal, or sphenoid electrode
9. Ictal EEG onset that is focal or lateralised on the ipsilateral side
10. Ipsilateral temporal focal hypometabolism on positron emission tomography (PET)
11. Consensus of ipsilateral mesial temporal origin based on a multidisciplinary discussion
12. Ability to understand and speak Mandarin

Exclusion criteria are as follows:

1. A history of serious cerebral insult after the age of 5
2. Progressive neurological disorders or mental retardation (I.Q. < 70)
3. Psychogenic seizures
4. Focal neurological deficits other than memory disturbances
5. Any unexplained focal or lateralised neurological deficits other than memory dysfunction
6. Temporal neocortical or extratemporal lesions on MRI
7. Psychosis, current or recent substance abuse, suicidality, or anorexia
8. Severe systemic disease
9. Unequivocal focal extratemporal EEG slowing or interictal spikes
10. Lesions outside of the mesial temporal area on MRI
11. Diffuse unilateral or bilateral hypometabolism on PET
12. Contralateral or extratemporal ictal onset
13. Persistent extratemporal/predominant contralateral focal interictal spikes or slowing; generalised interictal spikes
14. Inclusion in any clinical trial
15. Pregnancy

### Interventions

**Fig. 1:**
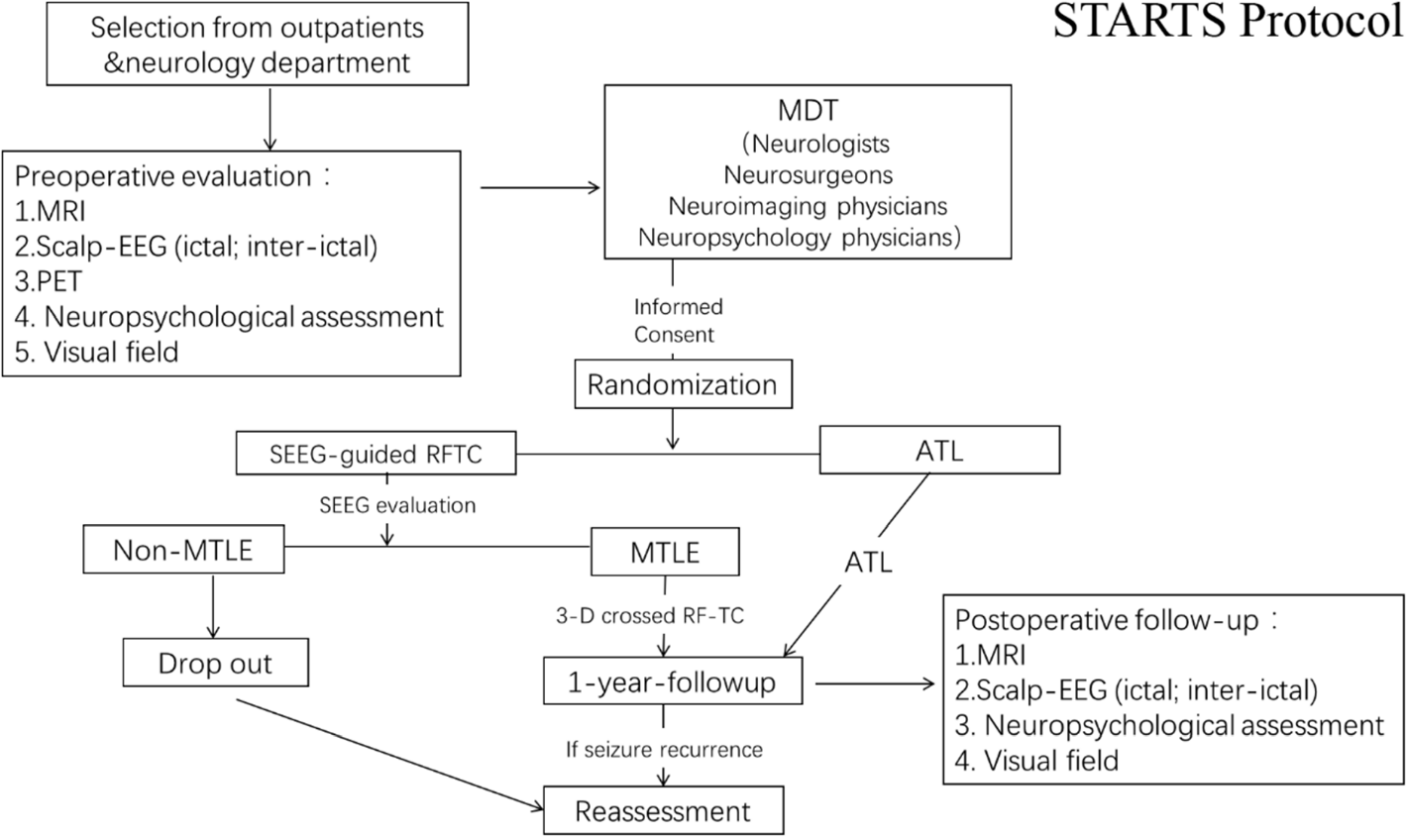
Timeline of the STARTS protocol. Patients assigned to the ATL group will undergo ATL directly. Patients assigned to the RF-TC group will undergo SEEG implantation and monitoring until the first seizure episode. Then, 3D-crossing RF-TC will be conducted

All participants will be randomised into two groups: an ATL group (arm 1) and an SEEG-guided RF-TC group (arm 2). The full technological flow of this RCT is shown in Fig. 1.

Patients enrolled in arm 1 will undergo open ATL following pre-surgical evaluation, which will be conducted as established by Wiebe et al. in their RCT for mesial temporal lobe epilepsy [4]. Two experienced neurosurgeons will perform ATL for mTLE and resection will be 5.5 cm on the non-dominant hemisphere or 4.5 cm on the dominant hemisphere, as referenced in Wiebe et al. [4]. The mesial part of the resection will include the ipsilateral amygdala, hippocampus, and uncinate gyrus. The full procedure will be recorded by the neurosurgeons performing the resection. Patients will continue taking the same AEDs that they had been taking prior to surgery under the supervision of a neurologist. Patients will not stop AED treatment until the 1-year follow-up even if they do not experience seizures.

**Fig. 2:**
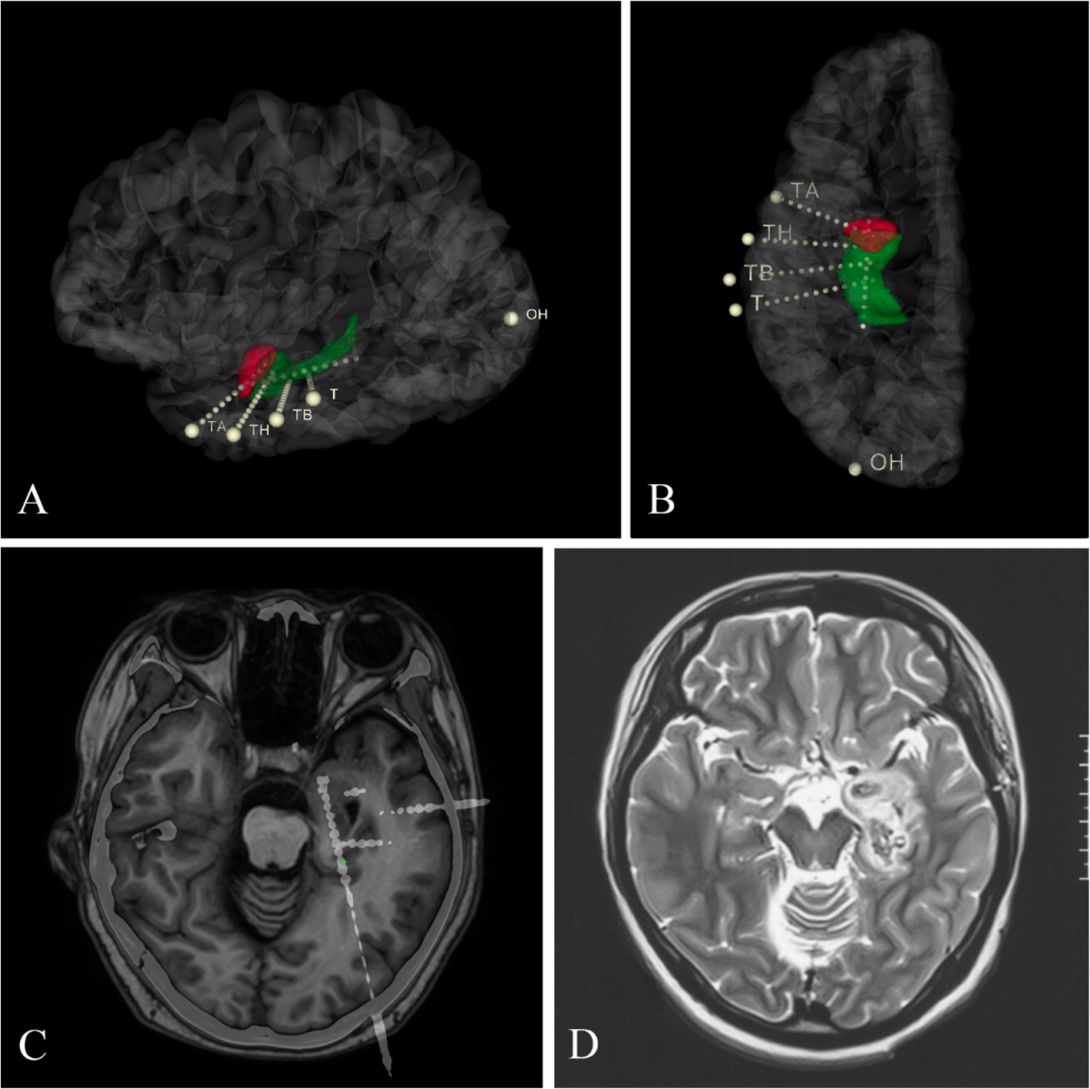
**A**, **B** The distribution of SEEG electrodes via three-dimensional reconstruction and the relationship between electrodes and the mesial temporal area. OH, T, TH, and TB were implanted in the hippocampus. TA was implanted in the amygdala. **C** The combination of preoperative T1-weighted images and postoperative CT images. The relative anatomical position of the SEEG electrodes and brain structure can be observed. **D** Postoperative T2-weighted MRIs obtained within 1 week of SEEG-guided RF-TC

Patients enrolled in arm 2 will undergo SEEG implantation and RF-TC for mTLE. SEEG implantation will be performed following pre-surgical evaluation. The target points of the SEEG electrodes will cover the mesial part of the ipsilateral temporal region. Both long-axis and parallel vertical depth electrodes generating a 3-dimensional space will be designed to guide RF-TC in cases of small ablation volume (Fig. 2). For each patient, bipolar coagulation will be performed on each of two contiguous contacts of the longitudinal and vertical electrodes using the same parameters. Contacts between electrodes will be further coagulated at intervals of < 5 mm to ensure freedom from vascular injury. This modified approach provides extended volume including the amygdala, hippocampus, subiculum, and part of the entorhinal cortex (Fig. 2). Similarly, patients will not stop taking AEDs until the 1-year follow-up.

Two doctors will store and manage the data entered in this study (Wei and Chen). If participants withdraw from this RCT, all the information will not be stored except the basic identity and consent form (information prior to the withdrawal).

### Outcomes

The primary outcome measure is cognitive function after treatment, which will be assessed using the Chinese version of the full-scale Wechsler Adult Intelligence Scale-IV (WAIS-IV-C) for adults (> 16 years old) or the Chinese version of the Wechsler Children’s Intelligence Scale-IV (WCIS-IV-C) for children. Assessments will be performed before and 1 year after surgery. Higher values are considered indicative of better outcomes.

Secondary outcomes are as follows:

1. Post-surgical seizure outcomes after 1 year based on Engel classification. Engel 1A and 1B will be considered to reflect seizure freedom, while Engel 1C–4 will be considered to reflect seizure recurrence. Seizure outcomes will be assessed at 3, 6, and 12 months via telephone interviews or outpatient visits with the patient and direct relatives. Seizures occurring within the first 2 weeks after surgery will not be considered to reflect seizure recurrence.
2. Visual field examination results: We will compare the number of patients with visual field defects between arm 1 and arm 2 before and 1 year after surgery.
3. The number of participants with procedure-related complications 1 year after surgery, including postoperative stroke with or without symptoms (via MRI), postoperative intracranial bleeding with or without symptoms (via MRI), postoperative intracranial infection, postoperative wound infection, and postoperative subcutaneous dropsy.
4. Quality of life after treatment. Values will be assessed using the Quality of Life in Epilepsy-89 scale (QOLIE-89) for adults (age 17–60) and the Quality of Life in Epilepsy-48 scale (QOLIE-48) for children (age 14–16) 1 year after surgery.
5. Average hospitalisation expenses 1 month after surgery.

All clinical and outcome-related data will be collected by two experienced clinicians (one neurologist and one neurosurgeon). Detailed data collection and follow-up timeline are shown in Table 1. For each patient, a case report form (CRF) including comprehensive patient information, clinical data, scale results, and outcomes will be completed.

**Table 1:** Data collection

Assessment	Baseline	Follow-up 7 ± 2 days	Follow-up 90 ± 7 days	Follow-up 180 ± 7 days	Follow-up 360 ± 14 days
Informed consent	□				
Demographics	□				
History of epilepsy	□				
Physical examination	□	□			□
EEG	□		*□		□
AEDs	□	□	□	□	□
Visual field assessment	□				□
WAIS-IV-C/WCIS-IV-C	□				□
QOLIE-89/QOLIE-48	□				□
Engel classification		□	□	□	□
PET	□				
MRI	□	□	*□		□
Adverse events		□	□	□	□
Concomitant medication	□	□	□	□	□

□, required; *□, optional; *EEG*, electroencephalogram; *AEDs*, antiepileptic drugs; *WAIS-IV-C*, Chinese version of the Wechsler Adult Intelligence Scale-IV; *WCIS-IV-C*, Chinese version of the Wechsler Children’s Intelligence Scale-IV; *QOLIE-89*, Quality of Life in Epilepsy-89; *QOLIE-48*, Quality of Life in Epilepsy-48; *PET*, positron emission tomography; *MRI*, magnetic resonance imaging

### Sample size

The sample size was calculated based on our previous experience (cognitive performance decrease of 25% after ATL), similar to findings reported in earlier studies [18]. Approximately 5% of patients exhibited decreased cognitive performance after SEEG-guided RF-TC in our previous series, while most previous studies have reported improvements or a lack of impairment [20–22]. We expect to enrol 20 patients in each arm using a one-sided 95% confidence interval, a non-inferiority limit of 10%, and an expected withdraw rate of 20%. Thus, we aim to include a total of 40 patients in this trial. Patients will be recruited from outpatient and MDT with strict inclusion criteria.

Participants will be replaced if they withdraw at any time prior to the final follow-up supervised by Dr Yong-Zhi Shan and Guo-Guang Zhao. Those who withdraw from the study during treatment because of specific medical or technical events will also be monitored and replaced. The trial conduct will be audited by the investigators with the whole MDT monthly.

### Randomisation and blinding

All patients and their direct relatives will be informed regarding the intentions and technological procedures involved in the study at the time of hospitalisation. Written informed consent will be obtained following patient agreement. The sealed envelope system will be used for randomisation of the patients into two groups. The envelope will be supported by a recognised third-party organisation. Neither the investigator nor the participants will be aware of each envelope’s contents.

Clinicians will know the exact allocation of each patient, and blinding will not be performed. In addition, patients will not be blinded due to the differences between the treatment strategies. Patients will also provide written informed consent the day prior to surgery.

### Statistical analysis

In the present study, we aim to compare treatment outcomes between two different surgical strategies for mTLE. Kaplan-Meier estimator will be conducted to compare the seizure outcome between two groups at 3, 6, and 12 months’ interval. Cognitive function, visual field defects, and Engel class I outcomes will be compared between the groups using χ^2^ tests or t tests. Subgroup analysis will be made if the results are positive. Average complication rate and hospitalisation expenses will be qualitatively compared between the two groups. For each separate group, a logistic analysis will be made to further explore the factors which may affect the outcomes (p < 0.05). Statistical analysis will be performed using SPSS version 21 (SPSS Inc., Chicago, IL, USA) and/or MATLAB_R2018a (MathWorks Inc., Natick, MA, USA).

### Patient and public involvement

We are here to state that the patients or public were not involved in the design of our research. If any harm exists, the health and surgery insurance will cover the indemnity to the patients. The protocol and the design of this study were discussed and developed by MDT including neurologists, neurosurgeons, neuroimaging physicians, and neuropsychology physicians.

### Ethical considerations and result dissemination

Written informed consent will be obtained from all participants and their representatives. The STARTS protocol has been approved by the ethics committee at Xuanwu Hospital and will be conducted in accordance with the Declaration of Helsinki. The data monitoring committee will consist of one neurosurgeon and two neurologists independent from this study. If there exists any modification, the changes should be submitted to the ethics committee and the study approval department. Unless approved, all the modifications should not be made. STARTS is also registered at the US National Institutes of Health (ClinicalTrials.gov: NCT03941613, registered on May 8, 2019). The schedule and the results of this study will be open on the ClinicalTrials.gov website and will also be open to peer-reviewed journals. If possible, the results will be shown at the national conference and will be further discussed accompany with specialists on the same research field. All information including identity, medical history, illness, medical examination, and laboratory results will be kept strictly confidential within the limits of the law. Unless authorised, the ethics committee and the study approval department may have access to the medical records related to this study to verify the authenticity and accuracy of the data collected from this study, with no personal details involving. All the participants’ personal information will be confidential to the public and journal.

## Discussion

RCTs are considered the most reliable form of scientific evidence given their ability to reduce spurious causality and bias. However, to our knowledge, the present study is the first RCT to compare cognitive outcomes and treatment results between SEEG-guided RF-TC and standard ATL for mTLE.

ATL has been considered an effective treatment for temporal lobe epilepsy and is now the most commonly used surgical technique for mTLE patients. Long-term seizure-free rates for ATL range from 59 to 80% [23]. However, previous studies have reported that ATL for mTLE is associated with complications such as memory impairments and visual field deficits [9, 20]. A systematic review by Sherman et al. noted verbal memory decline in up to 44% and 20% of patients undergoing dominant and non-dominant hemisphere ATL, respectively [24]. Given that SEEG-guided RF-TC may ensure better preservation of cognitive function and reduce the risk of visual field impairments, RCTs evaluating such treatment are required.

SEEG is a minimally invasive method for precisely localising the seizure-onset zone that is advantageous due to its ability for a three-dimensional definition of the epileptogenic zone [25]. Although SEEG-guided RF-TC has been used for typical cases of hypothalamic hamartoma, periventricular nodular heterotopia (PNH), and mTLE with or without hippocampal sclerosis, treatment outcomes have varied across studies, and the efficacy of RF-TC for mTLE remains controversial [13, 14, 16, 18]. Moles et al. compared postoperative seizure outcomes between SEEG-guided RF-TC and ATL, reporting that none of the patients in the SEEG-guided RF-TC group achieved seizure freedom. In contrast, 37 patients (75.5%) in the ATL group were seizure-free 12 months after treatment [12]. In a review of their series, Malikova et al. found that nearly 70% of patients were seizure-free postoperatively, similar to findings in our unpublished study [22]. Given these discrepancies, RCTs are also required to verify the non-inferiority of SEEG-guided RF-TC when compared with ATL.

Limitations of the study include the strict eligibility criteria which may lead the difficulty for the participant enrolment. Secondly, as we focused only on mTLE with hippocampal sclerosis patients, other types of temporal lobe epilepsy (such as neocortical temporal lobe epilepsy) will not be investigated in this study and further research may be promoted.

## Trial status

Recruitment ongoing (approximate recruitment completed date: February 28, 2021).

## Data Availability

The datasets used and/or analysed during the current study are available from the corresponding author on reasonable request.

## Abbreviations

EEG: Electroencephalogram
mTLE: Mesial temporal lobe epilepsy
ATL: Anterior temporal lobectomy
RF-TC: Radiofrequency thermocoagulation
SEEG: Stereotactic electroencephalography
RCT: Randomised controlled trial
MDT: Multidisciplinary team
AEDs: Antiepileptic drugs
I.Q.: Intelligence quotient
MRI: Magnetic resonance imaging
PET: Positron emission tomography
WAIS-IV-C: Wechsler Adult Intelligence Scale-IV
WCIS-IV-C: Wechsler Children’s Intelligence Scale-IV
QOLIE-89: Quality of Life in Epilepsy-89 scale
QOLIE-48: Quality of Life in Epilepsy-48 scale
CRF: Case report form
PNH: Periventricular nodular heterotopia

## References

[1] Krauss GL, Sperling MR (2011). Treating patients with medically resistant epilepsy. Neurol Clin Pract.

[2] Tatum WO (2012). Mesial temporal lobe epilepsy. J Clin Neurophysiol.

[3] Engel J (2001). Mesial temporal lobe epilepsy: what have we learned?. Neuroscientist.

[4] Wiebe S, Blume WT, Girvin JP, Eliasziw M (2001). A randomized, controlled trial of surgery for temporal-lobe epilepsy. N Engl J Med.

[5] W. S. Erkrankung des ammonshorns als aetiologisches moment der epilepsie. Arch Psychiatr Nervenkr 1880;10(3):631-675, DOI: 10.1007/BF02224538.

[6] Penfield W, Flanigin H (1950). Surgical therapy of temporal lobe seizures. AMA Arch Neurol Psychiatry.

[7] Engel J, McDermott MP, Wiebe S (2010). Design considerations for a multicenter randomized controlled trial of early surgery for mesial temporal lobe epilepsy. Epilepsia.

[8] Sagher O, Thawani JP, Etame AB, Gomez-Hassan DM (2012). Seizure outcomes and mesial resection volumes following selective amygdalohippocampectomy and temporal lobectomy. Neurosurg Focus.

[9] Quigg M, Barbaro NM, Ward MM, Chang EF, Broshek DK, Langfitt JT, Yan G, Laxer KD, Cole AJ, Sneed PK, Hess CP, Yu W, Newman SA, Mueller S, Tripathi M, Heck CN, Miller JW, Garcia PA, McEvoy A, Fountain NB, Salanova V, Knowlton RC, Bagić A, Henry T, Kapoor S, McKhann G, Palade AE, Reuber M, Tecoma E (2018). Visual field defects after radiosurgery versus temporal lobectomy for mesial temporal lobe epilepsy: findings of the ROSE trial. Seizure.

[10] Mathon B, Bielle F, Samson S, Plaisant O, Dupont S, Bertrand A, Miles R, Nguyen-Michel VH, Lambrecq V, Calderon-Garcidueñas AL, Duyckaerts C, Carpentier A, Baulac M, Cornu P, Adam C, Clemenceau S, Navarro V (2017). Predictive factors of long-term outcomes of surgery for mesial temporal lobe epilepsy associated with hippocampal sclerosis. Epilepsia.

[11] Marossero F, Ravagnati L, Sironi VA (1980). Late results of stereotactic radiofrequency lesions in epilepsy. Acta Neurochir Suppl (Wien).

[12] Moles A, Guenot M, Rheims S (2018). SEEG-guided radiofrequency coagulation (SEEG-guided RF-TC) versus anterior temporal lobectomy (ATL) in temporal lobe epilepsy. J Neurol.

[13] Lee CY, Li HT, Wu T, et al. Efficacy of limited hippocampal radiofrequency thermocoagulation for mesial temporal lobe epilepsy. J Neurosurg. 2018;131(3):781–9. 10.3171/2018.4.JNS184. 10.3171/2018.4.JNS184 30497199

[14] Cossu M, Fuschillo D, Casaceli G, Pelliccia V, Castana L, Mai R, Francione S, Sartori I, Gozzo F, Nobili L, Tassi L, Cardinale F, Lo Russo G (2015). Stereoelectroencephalography-guided radiofrequency thermocoagulation in the epileptogenic zone: a retrospective study on 89 cases. J Neurosurg.

[15] Abel TJ, Varela Osorio R, Amorim-Leite R, Mathieu F, Kahane P, Minotti L, Hoffmann D, Chabardes S (2018). Frameless robot-assisted stereoelectroencephalography in children: technical aspects and comparison with Talairach frame technique. J Neurosurg Pediatr.

[16] Wei PH, An Y, Fan XT, Wang YH, Yang YF, Ren LK, Shan YZ, Zhao GG (2018). Stereoelectroencephalography-guided radiofrequency thermocoagulation for hypothalamic hamartomas: preliminary evidence. World Neurosurg.

[17] Malikova H, Liscak R (2018). A neurosurgeon’s view: outcome after RF-ablation for mTLE. Epilepsy Res.

[18] Bourdillon P, Cucherat M, Isnard J, Ostrowsky-Coste K, Catenoix H, Guénot M, Rheims S (2018). Stereo-electroencephalography-guided radiofrequency thermocoagulation in patients with focal epilepsy: a systematic review and meta-analysis. Epilepsia.

[19] Vojtech Z, Malikova H, Kramska L (2014). Long-term seizure outcome after stereotactic amygdalohippocampectomy. Acta Neurochir (Wien).

[20] Witt JA, Hoppe C, Helmstaedter C (2018). Neuropsychologist’s (re-)view: resective versus ablative amygdalohippocampectomies. Epilepsy Res.

[21] Malikova H, Kramska L, Vojtech Z, Liscak R, Sroubek J, Lukavsky J, Druga R (2014). Different surgical approaches for mesial temporal epilepsy: resection extent, seizure, and neuropsychological outcomes. Stereotact Funct Neurosurg.

[22] Malikova H, Kramska L, Vojtech Z, Lukavsky J, Liscak R (2013). Stereotactic radiofrequency amygdalohippocampectomy: two years of good neuropsychological outcomes. Epilepsy Res.

[23] Tellez-Zenteno JF, Dhar R, Wiebe S (2005). Long-term seizure outcomes following epilepsy surgery: a systematic review and meta-analysis. Brain.

[24] Sherman EM, Wiebe S, Fay-McClymont TB (2011). Neuropsychological outcomes after epilepsy surgery: systematic review and pooled estimates. Epilepsia.

[25] Minotti L, Montavont A, Scholly J, Tyvaert L, Taussig D (2018). Indications and limits of stereoelectroencephalography (SEEG). Neurophysiol Clin.

